# Medical News from New York

**Published:** 1894-10

**Authors:** William Philip Spratling

**Affiliations:** New York


### *CORRESPONDENCE.*

---

### MEDICAL NEWS FROM NEW YORK.

NEW YORK, September 15, 1894.

The schools of medicine of this city are actively preparing to begin the session of '94 and '95. College professors are returning from vacations spent in Europe, at the seashore, and in the mountains; and first course men are beginning to be in evidence.

The College of Physicians and Surgeons (the Medical Department of Columbia College) opens its doors on Monday morning, October 1st. Every student who matriculates in that school for the first time this coming session, and who desires to secure a diploma from the same, will be obliged to take a four years' course in order to do so. The University here has increased the number of its courses from two to three, and begins the regular winter session October 3d. The session at Bellevue begins September 24th. Many schools South and West have likewise added a year to their curriculum. Why is this being done? Is it solely in the interest of higher medical education that those most interested in the welfare of the schools may feel to be a necessity? Perhaps so, but quite recently I heard a well known physician advance a different reason. He was of the opinion that the schools had to turn out a class of men better qualified to cope with the rigid examinations that are now held by the various county and State Examining Boards.

Coincident with the time increase required in the pursuit of medical studies, the fees demanded of students have grown proportionately. A few years ago, any young man who had a thousand dollars could, by practicing a little wholesome economy, take two courses of lectures, pay all his college fees, his board bill, buy books and get ready to enter practice. But to-day, if he studies at one of the best schools, he will require very nearly, if not quite that amount with which to pay his college fees alone.

The reported new remedy for diphtheria, antitoxine, which had its origin in Koch's laboratory in Berlin, does not appear to be creating a very great furor here. Dr. Biggs, who was sent abroad by the health authorities of this city to learn something about the new remedy, returned with a report based on its use in two hundred and fifty cases. The substance of Dr. Biggs's report was as follows: One hundred per cent. of cases got well when the inoculation was made during the first twenty-four hours of the disease. Ninety-seven per cent. of those inoculated on the second day got well. Eighty-seven per cent. got well when the inoculation was made on the third day. On the fourth seventy-six per cent., and when the inoculation was delayed until the fifth day, only fifty-seven per cent. recovered.

These figures represent an exceedingly favorable showing. If the presence of diphtheria be recognized during the first twenty-four hours, a person can be inoculated and at once rendered an immune. The *British Medical Journal* of August 18th contains reports of three well marked cases of diphtheria that were successfully treated with the new remedy. It is reported that Koch has been at work on the development, elaboration and testing of antitoxine for some years, and that he only gave the results of his labors to the public after he had thoroughly satisfied himself of its great value. Physicians everywhere will watch with keen yet conservative interest the crucial test of the new remedy,—its application in disease.

I will endeavor to send you reports of the first authentic cases that come to my knowledge in which it was used, with a report of results obtained.

A very feeble cholera scare agitated the atmosphere about the office of the health officer of the port a day or so ago. The Werra, an immigrant ship from Genoa, came in with six persons sick, all manifesting cholera symptoms. There was no illness on board until the ship had been five days out. The health officer caused the removal of the sick to Hoffman Island, where a bacteriological examination soon established the fact that they had only ordinary diarrhea. So much for bacteriological science. A few days ago in Cumberland, Md., a stranger, an immigrant recently landed sickened and died in a few hours. He manifested strong evidence of having died of genuine Asiatic cholera. The body was quickly buried and his companions were quarantined. The government had the body exhumed and set on foot a thorough bacteriological examination. A report was made that the man had died of ptomaïne poisoning generated in consequence of his having eaten bad food.

The problem of garbage disposal is agitating New York considerably just now. The practice of dumping the city's refuse out at sea, only to have it washed ashore again, and of trying to "build ground" with it by dumping it about and over a small island in the Sound, are both pernicious in the extreme. The favorable method of disposing of it up to a few months ago, was to carry it a few miles out to sea and dump it. This soon began to interfere with navigation, or threatened to do so by clogging the already too narrow channel through which vessels must pass to reach the city, and was stopped by the government. Then the city authoritie sought to enlarge the dimensions of a small island in the Sound opposite the upper portion of the city. A retaining wall of crib-work was constructed, and a putrescible and offensive mass was soon made there that covered over thirty acres. The stench that rose was horrible. Hundreds of persons living within range of the foul odors literally rose up in arms, held meetings, called on the mayor, sent a delegation to the governor, and finally sought the services of the grand jury. The court at last granted an injunction prohibiting any further dumping there. But the foul mass remained, driving people from their homes and causing sickness, so it is claimed. It was proposed to disinfect the entire mass. An electrician, Mr. A. E. Woolf, set up an electric plant on a barge by the side of the island and began to manufacture a very powerful and effective disinfectant from common sea-water. Four thousand gallons an hour were made, and by means of a hose, sprinkled over the entire area of the putrescible matter. This was kept up three weeks until the mass was saturated to a depth of about two feet. The bad odors were promptly stopped.

The process of making this fluid, "Electrozone" it is called, is exceedingly simple and by no means expensive. It consists simply in passing an electrical current through sea-water. This serves to transform the salt (chloride of sodium) into hypochlorate of sodium, which is a powerful disinfectant and deodorizer.

Meanwhile the problem of garbage disposal remains unsolved, and it is to be hoped that the commission named by the mayor to devise some adequate plan, will suggest something that will fill all requirements.

New York City is about to loose one of its best known medical landmarks. The New Bloomingdale Asylum, located at White Plains, twenty-five miles up the Hudson from this city, is about completed; and the transfer of patients from the famous old building here is now going on.

The new Bloomingdale will accommodate three hundred and fifty patients, which is fifty more than could be treated at one time in the old building.

The charter for this asylum was granted to the "Society of the Hospital of the City of New York" in 1770, by King George the Third. Subsequent legislation changed the name of the Society to the "Society of the New York Hospital."

Owing largely to its ramification here in the city, composed in the main of the New York Hospital on Fifteenth street, and Chambers Street Hospital, the new Bloomingdale will not be so quickly forgotten, even after we get accustomed to seeing the students of Columbia College safely domiciled within the historic walls of the old Bloomingdale Asylum.

WILLIAM PHILIP SPRATLING, M.D.

---

### BORO-SALICYLIC SOLUTION,

Introduced by Cesaris and Carcano, as an antiseptic, contains to every litre (say quart) four grammes (1 ℨ) each of boric and salicylic acids. Its therapeutic value has recently been tested in an Italian hospital and found superior to that even of corrosive sublimate. The addition of the boric acid renders the salycilic acid solution more permanent.—*Merck's Medical Report.*

